# The Influence of Dietary Interventions on Chronic Kidney Disease–Mineral and Bone Disorder (CKD-MBD)

**DOI:** 10.3390/nu13062065

**Published:** 2021-06-16

**Authors:** Jacek Rysz, Beata Franczyk, Robert Rokicki, Anna Gluba-Brzózka

**Affiliations:** 1 Department of Nephrology, Hypertension and Family Medicine, Medical University of Lodz, 90-549 Lodz, Poland; jacek.rysz@umed.lodz.pl (J.R.); bfranczyk-skora@wp.pl (B.F.); 2 Clinic of Hand Surgery, Medical University of Lodz, 90-549 Lodz, Poland; robert.rokicki@umed.lodz.pl

**Keywords:** chronic kidney disease, bone metabolism, secondary hyperparathyroidism, dietary interventions

## Abstract

Chronic kidney disease is a health problem whose prevalence is increasing worldwide. The kidney plays an important role in the metabolism of minerals and bone health and therefore, even at the early stages of CKD, disturbances in bone metabolism are observed. In the course of CKD, various bone turnover or mineralization disturbances can develop including adynamic hyperparathyroid, mixed renal bone disease, osteomalacia. The increased risk of fragility fractures is present at any age in these patients. Nutritional treatment of patients with advanced stages of CKD is aiming at prevention or correction of signs, symptoms of renal failure, avoidance of protein-energy wasting (PEW), delaying or prevention of the occurrence of mineral/bone disturbances, and delaying the start of dialysis. The results of studies suggest that progressive protein restriction is beneficial with the progression of renal insufficiency; however, other aspects of dietary management of CKD patients, including changes in sodium, phosphorus, and energy intake, as well as the source of protein and lipids (animal or plant origin) should also be considered carefully. Energy intake must cover patients’ energy requirement, in order to enable correct metabolic adaptation in the course of protein-restricted regimens and prevent negative nitrogen balance and protein-energy wasting.

## 1. Introduction

Chronic kidney disease (CKD) is a health problem whose prevalence is increasing worldwide [1]. According to estimations, 5–10% of the world population has CKD, as a result of the high prevalence of chronic diseases that are not well-controlled, such as arterial hypertension and diabetes mellitus [2]. Due to the fact that the kidney plays an important role in the metabolism of minerals and bone health, even at the early stages of CKD, disturbances in bone metabolism are observed and they progressively impair bone health [3,4]. Kidneys are the target organ of several hormones including parathormone (PTH) and fibroblast growth factor-23 (FGF-23), but also vitamin D is activated there [5]. In the course of chronic kidney disease, various bone turnover or mineralization disturbances can develop including adynamic, hyperparathyroid, mixed renal bone disease, osteomalacia, and osteoporosis; all of them may present with decreased bone mineral density (BMD) and/or be associated with fragility (including hip) fractures [6]. Changes in bone and mineral metabolism progress as kidney function declines [7]. In the early stages of CKD, CKD–mineral and bone disorder (CKD-MBD) is characterized by bone pain, bone fractures, skeletal deformities in growing children as well as decreased velocity in bone growth, abnormal height, and the calcification of vascular and other soft tissues [8,9]. Even asymptomatic individuals can present alterations in bone histology, such as osteitis fibrosa (characterized by high bone turnover due to hyperparathyroidism), adynamic bone disease (characterized by very low or lack of bone turnover frequently seen in patients with low PTH and low tissue-specific alkaline phosphatase activity) and osteomalacia (related to insufficient bone formation and mineralization) [6,10,11,12]. As the renal impairment aggravates, most patients develop more advanced bone and mineral metabolism disturbances, including renal osteodystrophy (ROD) manifested with bone pain, muscle-tendon rupture, pruritus, and increased incidence of fractures and CKD–mineral and bone disorder (CKD-MBD), which describes a systemic disorder of mineral and bone metabolism due to CKD. The results of studies have demonstrated low bone mineral density (BMD) in patients with CKD stages 3a–5D and 1.5- to 2-fold higher risk of fractures than in the general population and in BMD-matched patients without CKD [6,13,14,15,16]. According to studies, CKD-MBD is a substantial contributor to diminished quality of life and enhanced risk of mortality and morbidity in this group of patients [9,17,18]. The results of observational studies have shown the relationship between abnormal levels of CKD-MBD markers and poor clinical outcomes in pre-dialysis and dialysis patients [19,20]. The presence of high phosphate and calcium levels accompanied by high or low PTH levels was associated with greater mortality in maintenance hemodialysis patients [21]. Many studies have indicated that higher levels of alkaline phosphatase correlated with amplified risk of hospitalization and death in patients with CKD stages 3–4 and HD [22,23]. Alkaline phosphatases play important role in the pathogenesis of vascular calcification, which could explain why its high levels are associated with increased mortality. Other studies have revealed a strong correlation between FGF23 levels and poor renal outcomes [24,25,26]. The studies of the impact of elevated FGF23 levels in CKD patients on all-cause and cardiovascular mortality provided conflicting results. Some of them revealed the relationship between high levels of FGF23 and increased mortality [26,27,28], while others found no significant association [29,30].

The main aim of this review is to present the issue of chronic kidney disease–mineral and bone disorder (CKD-MBD) and to summarize the results of numerous studies assessing the impact of various diets on its course in order to imply the best choice of nutritional intervention. The selection of articles for this literature review was based on a PubMed search (the terms “CKD-MBD” + “pathomechanism” + “diet” + “CKD” were applied). We focused mainly on randomized, controlled studies/trials and systematic reviews and meta-analyses.

## 2. Pathogenesis of Chronic Kidney Disease–Mineral and Bone Disorder (CKD-MBD)

The mechanisms underlying bone loss and fractures in CKD patients are complex and have not been completely understood. According to the definition contained in a position statement from Kidney Disease: Improving Global Outcomes (KDIGO) [17] chronic kidney disease–mineral and bone disorder (CKD-MBD) is a “systemic disorder of mineral and bone metabolism due to CKD manifested by either one or a combination of the following: abnormalities of calcium, phosphorus, PTH, or vitamin D metabolism, abnormalities in bone turnover, mineralization, volume, linear growth, or strength; or vascular or other soft tissue calcification”.

The worsening of renal function is associated with the retention of phosphate resulting in hypocalcemia, hyperphosphatemia, and low 1,25(OH)2D3, all promoting parathyroid hormone (PTH) secretion which in consequence leads to increased phosphate excretion and the development of secondary hyperparathyroidism in advanced stages of CKD [1,18]. The mechanism enabling the counterbalancing of phosphate retention involves the enhanced production of fibroblast growth factor 23 (FGF-23) whose level rises very early in CKD. FGF-23 factor is derived from osteocytes and osteoblasts, and it is involved in direct bone–kidney and bone–parathyroid associations (e.g., the metabolism of vitamin D and phosphate) thus participating in the development of CKD-MBD [31].

Plasma FGF23 has been shown to stimulate the secretion of phosphate in the proximal renal tubule as a result of the downregulation of luminal sodium-dependent phosphate transporters expression. Also, it can reduce intestinal phosphate absorption via the inhibition of the NaPi cotransporter activity [32]. FGF23 was found to increase distal sodium reabsorption via the direct regulation of the thiazide-sensitive sodium-chloride transporter (NCC) level in the distal convoluted tubule as well as to stimulate calcium reabsorption via the activation of the apical calcium entry channel (transient receptor potential cation channel subfamily V member 5; TRPV5) in the distal tubule [33,34]. In the early stages of CKD, FGF23 down-regulates the activity of 1α-hydroxylase and enhances 24-hydroxylase activity which results in the decreased synthesis of 1,25-dihydroxyvitamin D [1,25(OH)2D3] [35,36]. Plasma levels of FGF23 gradually increase along with the worsening of renal function. Its secretion occurs earlier than changes in calcium, phosphorus, or PTH levels and therefore it is now recognized as one of the earliest detectable biomarkers of the CKD-MBD [37]. High levels of FGF23 diminish hyperphosphatemia reducing at the same time 1,25(OH)2 vitamin D levels [36]. The deficiency of calcitriol is associated with reduced intestinal calcium absorption, which results in hypocalcemia, decreases tissue levels of vitamin D receptors leading to the development of resistance to calcitriol-mediated regulation, the stimulation of PTH secretion and consequent secondary hyperparathyroidism [38]. The aggravation of renal injury and the related drop in the number of functioning nephrons, the maintenance of phosphate excretion is associated with FGF23 and PTH-stimulated decrease in tubular reabsorption of filtered phosphate in the remaining nephrons [39]. Due to the fact that Klotho deficiency limits the impact of FGF23 on phosphate excretion, PTH becomes the vital adaptive mechanism preserving phosphate homeostasis. Indeed, in dialysis patients, the ability of FGF23 to control phosphate levels via its phosphaturic effect as well as to normalize PTH secretion was demonstrated to be significantly reduced. In advanced CKD (stages 4–5), the aforementioned adaptation mechanism is no longer sufficient, and hyperphosphatemia develops in spite of high PTH and FGF23 levels [39].

The endocrine activity of FGF-23 requires its binding and the activation of its receptor complex containing a transmembrane protein Klotho [40,41]. The results of studies have indicated that the dysregulation of the FGF23-related compensatory mechanism in CKD patients is associated with Klotho deficiency [1]. In the early stages of CKD, the FGF23-Klotho axis was found imbalanced—high levels of FGF23 and reduced concentrations of Klotho were observed [1]. Apart from converting FGFR1(IIIc) into a specific receptor for FGF23, Klotho increases phosphaturia and prevents urinary calcium loss [42]. The decrease in the membrane-bound Klotho expression limits of FGF23-related signal transduction mediated by the FGF receptor/Klotho complexes leads to the loss of negative feedback in response to FGF23 secretion and subsequent persistent production and secretion of FGF23 by the osteocyte [43]. Its deficiency is, therefore, associated with mineral metabolism disorders, vascular calcifications, secondary hyperparathyroidism, and cardiac hypertrophy [40]. The increase in serum PTH observed from the early stages of CKD leads to enhanced bone resorption to maintain calcium balance. Prolonged hypersecretion of PTH is associated with high turnover bone disease and a greater risk of fractures and bone deformities [44]. Elevated serum phosphate has been demonstrated to reduce calcitriol synthesis and to precipitate in vessels and soft tissues with calcium, thus diminishing ionized calcium fraction and indirectly promoting PTH secretion [44]. Excessive PTH secretion associated with the reduction in serum 1,25(OH)2D3 and subsequent decrease in intestinal calcium absorption, as well as with low concentration of calcitriol and hyperphosphatemia leads to the mobilization of calcium from the bone and osteitis fibrosa [1,45]. Hyperphosphatemia promotes the transition of osteoblasts in the vessels stimulating extraskeletal mineralization and rising Ca × P product [39]. Increased levels of PTH are also associated with abnormal osteoblastic function and osteocyte stimulation with the receptor activator of NF-κB (RANK) ligand (RANK-L) leading to CKD mineralization defects, high bone turnover ROD, and bone resorption [46]. In CKD patients, the upregulation of RANK-L is accompanied by the downregulation of the osteoclast maturation inhibitor, osteoprotegerin (which protects from bone resorption), which in consequence favors bone resorption and increased bone turnover [47]. Numerous studies have demonstrated that the presence of kidney disease is associated with the reactivation of developmental programs involved in nephrogenesis during disease-stimulated renal repair, especially Wnt inhibitors (a portmanteau of wingless and int) such as Dickkopf-1 (Dkk1) and sclerotin [43,48,49]. These signal molecules are typically silent in the normal adult kidney [50]. The reactivation of the Wnt pathway regulating tubular epithelial proliferation and polarity during nephrogenesis is a driving force in renal fibrosis [48]. Wnt/ß-catenin pathway is considered to be one of the key regulators of bone formation since its activation stabilizes ß-catenin—a transcription factor involved in the synthesis of many osteoblastic factors, including Runx2 and osterix [51]. ß-catenin translocation to the nucleus becomes a triggering factor for osteoblastic genes and bone differentiation. Its activation enhances osteoblastic activity and intensifies bone formation [51]. It has been revealed that kidney disease-induced “unintended” systemic inhibition of Wnt activity is associated with severe consequences in the skeleton and vasculature, such as decreased bone formation rates, elevated FGF23 secretion, vascular calcification, and promotion of cardiac hypertrophy [43,50]. According to studies, activin A, belonging to TGFβ superfamily members, also plays a crucial role in the vascular and skeletal components of CKD-MBD [52]. It is secreted from the peritubular myofibroblast of injured kidneys and exerts its actions via type 2 activin A receptor (ActRIIA) [1,52]. Studies on animal models demonstrated that CKD-MBD, apart from elevated levels of FGF23, PTH, reduced Klotho hyperphosphatemia, osteodystrophy, vascular calcification, and cardiac hypertrophy, was characterized by the stimulation of ActRIIA. Moreover, the inhibition of ActRIIA signaling was associated with the reversal and attenuation of these features of CKD-MBD [52,53,54]. Recent studies have suggested that other factors may also modulate osteoblast function and may be involved in the development of the mineralization disorder of CKD thus leading to high turnover renal osteodystrophy, surplus bone resorption, skeletal frailty, and increased fracture risk [43,55]. The loss of bone strength despite an apparent increase in mass may be partly associated with the deposition of woven immature collagen fibrils instead of lamellar mature fibrils in the course of progressive renal impairment [56]. Overall bone balance determines whether patients with advanced CKD would experience gain or loss in bone volume [43]. Negative bone balance is associated with cortical and cancellous bone loss occurs which leads to osteopenia or osteoporosis [57]. In the case of positive bone balance, patients show osteosclerosis resulting from deposition composed primarily of immature woven collagen in new bones by osteoblasts [43]. However, the improvement of secondary hyperparathyroidism treatment nearly has ruled out this scenario. High-turnover renal osteodystrophy, observed in patients with secondary hyperparathyroidism with osteitis fibrosa, is characterized by bone resorption rates that exceed bone formation as well as osteopenia progressing to osteoporosis [58]. In turn, low-turnover renal osteodystrophy, diagnosed in those with over-treated secondary hyperparathyroidism is associated with decreased bone formation and resorption rates despite the fact that resorption is still in relative excess and loss of bone mass [59]. Patients with renal osteodystrophy are predisposed to cardiovascular calcification resulting in their higher morbidity and mortality [17,60].

Chronic kidney disease also leads to the significantly decreased ability to generate new bicarbonate required to buffer the amount of acid generated by the tissues, which results in acidosis [61,62]. Chronic metabolic acidosis occurs early in the course of CKD. It affects bone fragility via enhanced secretion and resistance to PTH, higher osteoclastic activity, raised renal elimination of calcium, bone mineral dissolution, and malnutrition [7,61,63]. The accumulation of uremic toxins has been proven to influence bone quality in CKD patients since they affect bone metabolism and function by worsening bone quality and quantity [64]. Indoxyl sulfate (IS), whose level increases as renal dysfunction progresses, down-regulates the expression of PTH receptor on osteoblasts and reduces the bone turnover as well as suppresses bone formation signaling by stimulating the secretion of Wnt antagonists from osteocyte [6,65,66]. Barreto et al. [67] found a positive correlation between IS levels and bone formation rate, osteoblasts surface area, osteoid volume, and bone fibrosis volume. Therefore, it seems that the decrease of gastrointestinal absorption and the increase in uremic toxin clearance may be important in the treatment of uremic osteoporosis.

## 3. Diets

In order to prevent the development of CKD-MBD, bone complication patients, as well as vascular or other soft tissue calcification CKD patients, should be offered not only treatment but also appropriate nutritional counseling [9]. Current research indicates that phosphate retention plays a significant role in the development of CKD-MBD. It seems that the control of phosphate homeostasis early in CKD may help to diminish adverse clinical consequences of mineral and bone disorders. Dietary phosphate restriction and the use of phosphate binders are two basic measures for the management of increased phosphate levels. Dietary interventions seem to be uncomplicated, inexpensive, and practical in comparison with drug therapies. The results of studies suggest that prolonged limiting of dietary phosphate intake can effectively prevent the development of secondary hyperparathyroidism, and this approach can be implemented in patients with all stages of kidney disease [68,69]. Therefore, dietary phosphate restriction is recommended in many guidelines. According to the KDOQI 2003 guidelines, dietary phosphorus should be restricted to 800–1000 mg/day in CKD patients with plasma levels of intact PTH exceeding the target range of the CKD stage [70]. The KDIGO 2009 guidelines suggest that dietary phosphate intake should be limited in patients with CKD stages 3 to 5D in order to treat hyperphosphataemia; however, the evidence to support this recommendation is currently weak [9,71].

### 3.1. Low Phosphate Diet/PPR Diet

The systematic review of randomized controlled trials revealed that serum phosphorus was significantly decreased in patients on a low phosphorus intake diet compared to a normal diet (MD −0.18 mmol/L, 95% CI −0.29 to −0.07; I2 = 0%) [9]. In turn, Isakova et al. [72] demonstrated considerably lower concentrations of PTH with low phosphorous intake compared to ad libitum diet (MD 25.60 pg/mL, 95% CI 5.13 to 46.07). However, phosphate restriction with the addition of a binder seems to be better than the restriction alone. A randomized controlled trial of patients with CKD stages 3–4 and normal serum phosphate levels showed that consumption of low-phosphorous products and the addition of phosphate binders were more efficient in the reduction of serum FGF-23 than either approach alone [72,73]. Other systematic review and meta-analyses assessing the effect of phosphate-specific diet therapy on serum phosphate in patients undergoing HD revealed that monthly diet-therapy sessions (lasting approximately 20–30 min) could considerably decrease serum phosphate in adults on HD with hyperphosphatemia over a 6-month period (two RCTs [74,75]), while a single session of diet therapy could reduce serum phosphate in adults with hyperphosphatemia for up to 3 months (one RCT [76]) [77]. The authors suggested that the impact of diet on serum phosphate could be associated with diminished dietary phosphate load. Patients on phosphate-specific diets claimed to adhere to specific strategies involving the avoidance of phosphate additives and limitation of phosphate-rich foods intake [77]. Dietary phosphate restriction is used commonly to improve outcomes, but the fact that phosphate intake usually parallels protein intake makes the situation more complicated as CKD patients need to receive an adequate amount of dietary protein to avoid malnutrition [78]. Muscular wasting CKD has been observed in the course of CKD and therefore there are concerns about potential catabolic and cachectic effects of LPD/VLPD. Therefore, recently an approach has been offered that considers the phosphate to protein ratio PPR (the phosphorus content per gram of protein) [79]. Such a strategy has several benefits as dietary management for CKD patients with hyperphosphatemia. Firstly, it concentrates concurrently on both proteins and phosphates, which are vital for the nutritional treatment of CDK, and it is independent of the portion size or serving [79]. Moreover, the calculated ratio enables patients to select from two similar options with different amounts of phosphorus but almost equal amounts of protein [80]. The utilization of this approach allows for the maintenance of proper protein intake including foods with low PPR, avoidance of a high phosphate load, and also the appropriate inclusion of low phosphate vegetables, fruits, and cereals into the diet [79].

### 3.2. Low-Protein Diets and Supplemented Very-Low-Protein Diets

According to studies, patients with advanced CKD (stages 4 and 5) who are on reduced phosphate and protein intake diet show improved short-term control of secondary hyperparathyroidism [81,82,83]. The adjustment of dietary protein intake is a cornerstone of nutrition therapy in patients with CKD [84,85]. The recommendation often varies between very-low (VLPD) diets containing 0.3–0.4 g of protein/kg/d supplemented with alpha-keto acid (KA) analogues of essential amino acids and low-protein diets (LPD) comprising 0.6–0.8 g protein/kg/d and usually no KA supplementation [86,87]. The analysis of NHANES data revealed that dietary protein intake observed in most stages of pre-dialysis CKD patients exceeded recommended dietary allowance (RDA) (0.8 g/kg/day of protein) [88]. To facilitate the decrease of protein intake, Fois et al. [89] suggested a stepwise approach involving the preliminary reduction of dietary protein intake to RDA followed by the transition into LPD within a 2- to 6-week period and ending with the switching to (if desired) VLPD + KA analog diet. Numerous studies have indicated that LPD exert beneficial effects on markers of CKD-MBD, especially when it is combined with KA supplementation [85].

In the long perspective, reduction of phosphate and protein intake allowed some patients to accomplish a normal rate of bone formation [90]. Another study indicated that intensive restriction of protein and phosphate intake reduced FGF-23 and serum phosphorus levels compared to a low-protein diet [91]. A secondary analysis of the Modification of Diet in Renal Disease (MDRD) study revealed that a lower protein intake (0.87 g protein/kg/day) was associated with a slight reduction in serum phosphorus that was sustained over a three-year period compared to standard protein intake (1.12 g protein/kg/day) [92].

In turn, Liu et al. [9] demonstrated that very low protein intake was more effective in diminishing PTH level compared to low protein intake (MD −69.64 pmol/L, 95% CI −139.83 to 0.54; I2 = 57%). However, they also observed that very low protein intake was not superior to conventional low protein intake in terms of impact on serum phosphorus (MD −0.12 mmol/L, 95% CI −0.50 to 0.25), serum calcium (MD 0.00 mmol/L, 95% CI −0.17 to 0.17), or alkaline phosphatase (MD −22.00 U/L, 95% CI −78.25 to 34.25) [9]. Moreover, Herselman et al. [93] found that very low protein intake (0.4 g protein/kg/d) and low protein intake (0.6 g protein/kg/d) (MD 0.00 mmol/L, 95% CI −0.17 to 0.17) did not significantly influence body mass index, arm muscle area, percentage body fat. They also had no effect on serum levels of parathyroid hormone and alkaline phosphatase, tubular reabsorption of phosphate, and the theoretical renal threshold for phosphate. The effect of various contents of protein in the diet on bone mineral density (BMD) or T scores (*p* < 0.0001) was also analyzed by Lee et al. [94] in 12,812 patients from the National Health and Nutrition Examination Survey database. In their analysis, the highest bone BMD and T scores were reported in the group with the highest protein intake (*p* < 0.0001) [95]. In case of femoral BMD, higher protein diets were associated with higher BMD in the femoral neck, trochanter, intertrochanteric, and total femoral areas (*p* = 0.032, 0.0036, 0.008, and 0.0039, respectively) but only in patients without CKD [94]. Therefore, it seems that diets containing higher amounts of protein result in higher femoral BMD only in subjects without CKD. In general, it seems that LPD may prove beneficial in mineral and bone metabolism compared to normal- or high-protein diets due to related dietary phosphorus intake [95].

The results of some recent studies have suggested that restricted very-low protein diets (VLDP), supplemented with ketoacid analogs (KAs) may not only improve renal function, but also prevent hyperparathyroidism, the accumulation of uremic retention solutes and insulin resistance [96]. It remains unknown whether KA supplementation alone exerts any beneficial effects on metabolic disturbances related to CKD. Randomized controlled trial of non-diabetic CKD 3b-4 stage patients ascribed to either LPD diet (0.6 g/kg of body weight/day) alone or with the supplementation of KA and demonstrated that the latter diet considerably diminished serum phosphorus, PTH, and FGF23 and increased serum Klotho in patients [97]. Lineadeau et al. [98] demonstrated an improvement in osteofibrotic and osteomalacic changes on bone biopsies after 12 months of treatment with KAs, which was independent of calcium intake. In turn, the study of patients with early (eGFR >  60 mL/min/1.73 m^2^) and advanced stages of CKD (< 30 mL/min/1.73 m^2^) on LPD demonstrated that this diet reduced intact FGF23 levels in both groups, while the decrease in serum phosphorus and PTH was seen only in advanced CKD [99]. KA supplementation can be used in combination with an LPD; however, it is most commonly prescribed with a VPLD [85]. There are several randomized controlled trials evaluating the impact of VLPD + KA on biochemical markers of CKD-MBD. The meta-analysis of 17 RCTs and 1459 participants assessing the safety and effectiveness of a restricted protein diet supplemented with KAs compared with regular diet or low protein diet (LPD) without KAs in CKD patients revealed that the first one not only preserved eGFR but also decreased proteinuria, serum phosphate, parathyroid hormone (PTH) level [100]. Only VLPD with KAs significantly improved serum PTH, while only LPD with KAs significantly increased serum albumin and serum calcium [100]. A systematic review and meta-analysis of five clinical trials assessing the effects of VLPD + KA in dialysis ESKD patients indicated a reduction in serum phosphorus of −1.14 mg/dl (95% CI −1.98, − 0.28) and a mean decrease in PTH of −212.35 ng/mL (95% CI −294.28, −130.42) with VLPD + KA [101]. The comparison of VLPD + KA (for 3 months) and Mediterranean-based diet or usual diet in moderate staged CKD patients (stages 3b–4; eGFR 15–45 mL/min/1.73 m^2^) revealed that the first diet was associated with a decrease in serum phosphorus and PTH as well as lowered FGF23 levels [91,102]. However, some other studies found that very low protein intake was not superior to the conventional low protein diet in terms of the effect on serum calcium, phosphorus, and alkaline phosphatase levels [103,104]. Low protein intake supplemented with keto-acids was shown to decrease serum phosphorus in patients undergoing hemodialysis compared with normal protein intake [104]. However, no differences in PTH and alkaline phosphatase were observed when hemodialysis patients adopted a hypolipidemic diet compared with statins [105]. Chauveau et al. [106] demonstrated that VLPD + KA may also exert an effect on bone mineral density. In advanced-staged patients, pre-dialysis CKD patients (eGFR 15  ±  4.7 mL/min/1.73 m^2^) the consumption of VLPD + KA for years induced initial decline in lean body mass (after 3 months) followed by stabilization at 6 months and then a significant increase from 6 to 24 months (*p* = 0.02, paired t-test). Moreover, they observed a significant reduction in total bone mass, lumbar or hip site bone mass, and Z-score from T0 to 1 and 2 years (*p* < 0.05). However, more studies are required to evaluate the direct effect of LPD and VLPD on bone outcomes in CKD patients. In general, many trials have demonstrated that VLPD + KA may beneficially affect biochemical markers of CKD-MBD. However, due to the fact that KA is provided in the form of calcium salts, there is a potentially increased risk for calcium retention related to high calcium burden [107,108]. Dietary protein reduction has been shown to diminish the risk of adverse outcomes related to high phosphate diets, such as renal osteodystrophy, left ventricular hypertrophy, and the progression of CKD [73]. Some studies have suggested that it can also help to decrease elevated FGF-23 levels which are associated with enhanced cardiovascular events and mortality risk [26,109].

### 3.3. High-Protein Diets in CKD and ESKD

In contrast to pre-dialysis CKD patients who are recommended dietary protein restrictions, a high protein intake is suggested for patients with dialysis ESKD in order to make up for the increased protein requirements, and protein losses during dialysis [84]. According to guidelines, HD patients are recommended to consume 1.2 g of protein/kg/d, while protein intake of patients on peritoneal dialysis should be in the range of 1.2–1.3 g of protein/kg/d to 1.5 g/kg/d (in case of peritonitis) [84,86]. Dietary protein has been demonstrated to exert beneficial effects on bone health due to the fact that it elevates the level of insulin-like growth factor 1 (IGF-1) which enhances the activity of osteoblasts and fractional calcium absorption, decreasing at the same time osteoclast activity [85,110]. Recent meta-analyses have confirmed the relationship between higher dietary intake of protein and higher total bone mineral density in healthy individuals [111]. A post-hoc analysis of the IHOPE trial revealed that 12-month supplementation of 30 g of whey protein with or without intradialytic bicycling prevented the yearly decrease in total and hip bone mineral density in elderly individuals [112]. On the other hand, higher dietary protein can increase endogenous acid production and net acid excretion which in patients with already compromised acid-base balance as a result of kidney dysfunction may negatively influence bone health [113]. Moreover, a high protein content diet is strongly associated with a high phosphorus intake which may also not be beneficial in this group of patients [79]. However, there are hardly any studies on the effect of higher dietary protein intake on biochemical markers of mineral and bone metabolism, bone, and vascular calcification in dialysis patients thus it is difficult to draw any conclusions.

### 3.4. Plant-Based Diets

Recent evidence implies that the source of phosphates in the diet (plant- vs. animal-based food), not only their content, is of importance [114]. Plant-based diets have been demonstrated to be advantageous as they help to reduce risk factors for many chronic diseases, including CKD [85]. Being on such a diet is associated with lower consumption of proteins and thus phosphorus and sulfur-containing amino acids and acid load [115]. The correction of potential metabolic acidosis in CKD patients resulting from diminished acid load translates to benefits to bones [85,116,117]. Goraya et al. [118] indicated that increasing consumption of fruits and vegetables improved (within a year) markers metabolic acidosis in moderate CKD patients. Plant-derived dietary phosphates are mostly in the form of less digestible (in humans) phytates and therefore, after their intake, they are less bioavailable compared to phosphates derived from animal sources [114]. A small study of patients with diagnosed CKD (mean eGFR 32 mL/min/1.73 m^2^) demonstrated that after one week of the dietary intervention (consumption of vegetarian- vs. meat-based diets with equivalent contents of protein and phosphates) the vegetarian diet resulted in lower serum levels of phosphorus and FGF-23 compared to meat-based diet [119]. In turn, Moorthi et al. [120] observed that the conversion from an animal protein-based diet to a 70% plant-protein diet was associated with a significant reduction of urinary phosphorous excretion, but not serum phosphorous or FGF-23 levels in patients with stages 3–5 CKD. Soroka et al. [121] analyzed the effects of low phosphate diets and found that a low-phosphorus vegan diet containing only an appropriate cereal-legume mixture was equally effective as a conventional low-protein diet. In their study, patients not only avoided protein malnutrition but also decreased phosphate intake despite its abundance in animal-based foods [9]. On the other hand, higher phytate intake may lead to deficiencies of some minerals such as iron, zinc, and calcium [79]. However, further studies of plant-based diets in CKD are required to analyze their beneficial effects in terms of bone health.

### 3.5. Other Approaches

Some studies have suggested the existence of a relationship between obesity, energy-dense diets, and mineral metabolism in CKD [122]. According to them, energy-dense food generally tends to contain low levels of Ca and also to decrease its intestinal absorption as a result of the interaction between Ca and lipids in the intestinal lumen and subsequent formation of Ca soaps [123,124]. Therefore, being on energy-dense diets may lead to negative Ca balance, and ultimately aggravate hypocalcemia in patients with CKD-MBD. Moreover, such products are typically rich in inorganic P (food additive) which is more easily absorbed compared with the organic P naturally contained in food [125]. Again, high-fat content in the diet enhances P digestibility, further aggravating P load [126]. The aforementioned trapping of Ca in Ca soaps reduces the formation of insoluble Ca–P complexes, thus allowing for the absorption of higher levels of P ions [126]. Data from animal studies seem to confirm that energy-dense diets could contribute to P retention in individuals with decreased renal function. Phosphorus overload is especially harmful for CKD-MBD patients, due to the risk of adverse consequences, including soft tissue mineralization and the progression of renal disease. Therefore, apart from malnourished individuals, energy-dense diets are inadequate for CKD-MBD patients, due to their impact on P metabolism. The above-mentioned energy-dense food-related decrease in Ca load and elevation of P load could exert a direct impact on parathyroid glands, leading to the increased secretion of PTH as well indirectly affecting PTH concentration via the promotion of hyperlipidemia and hyperleptinemia. The ingestion of a high calorie/high-fat diet has been also shown to increase plasma concentrations of FGF23 and the effect was independent of obesity [127,128]. It seems that the mechanism for increased FGF23 following the intake of energy-dense diets may be associated with the decrease in renal Klotho and subsequent tubular resistance to FGF23. At that time more FGF23 is required to maintain phosphaturia, which, in consequence, leads to the raise in circulating levels of FGF23 [129]. Moreover, high content of fat and obesity may stimulate systemic inflammation and renal injury which could also affect FGF23 [130,131].

However, currently, international guidelines failed to reach a complete agreement regarding the optimal amount of dietary proteins for CKD patients. A randomized controlled trial provided preliminary evidence that in CKD patients a protein intake of 0.55 g/kg/day guaranteed a better metabolic control and a decreased need of drugs, without a substantial risk of malnutrition, compared with a 0.8 g/kg/day intake [132].

The most important results obtained in the aforementioned studies concerning the impact of dietary interventions on CKD-MBD are summarized in Table 1.

## 4. Management of CKD-MBD

KDIGO guidelines recommended annual control of one of the markers of high bone turnover—alkaline phosphatase—as such tests are relatively cheap and help to identify patients at risk of higher mortality [23,133]. According to the 2009 KDIGO guidelines, the monitoring of serum levels of calcium, phosphate, PTH, and ALP should be performed starting from CKD [71]. Moreover, in those in CKD stages 4-5D, ALP should be measured at least once every 12 months if PTH levels are elevated. Due to the fact that in CKD patients and those with adynamic bone disease, the use of anti-resorptive agents is not recommended as they worsen microdamage accumulation leading to fractures, it is highly important to differentiate between patients with a high and low bone turnover state (based on bone turnover markers) and to monitor turnover status periodically in order to titrate therapy [134]. The measurement of serum PTH or bone-specific alkaline phosphatase (BSAP) should be carried out in patients with CKD 3-5D in order to evaluate bone disease. Significantly high or low values predict underlying bone turnover (2B) [71]. According to studies, plasma bone-specific alkaline phosphatase (bAP) appears to be more reliable in diagnosing high-turnover bone disease in patients with metabolic bone diseases compared to total alkaline phosphatases (tAP) [135]. The measurement of bone turnover markers enables not only the assessment of fracture risk but also might help to predict vascular calcification in CKD. Two such biomarkers—one bone formation marker (serum procollagen type I N propeptide, s-PINP) and one bone resorption marker (serum C-terminal cross-linking telopeptide of type I collagen, s-CTX)—were recommended by the International Osteoporosis Foundation (IOF) and the International Federation of Clinical Chemistry and Laboratory Medicine (IFCC) Working Group on Bone Marker Standards (WG-BMS) to be used as reference markers in these patients [134]. Among other markers which are unaffected in CKD, there are the tartrate-resistant acid phosphatase (TRAP) 5b and the bone-specific alkaline phosphatase (BSAP). These two markers have been shown to correlate well with bone formation rate [135]. However, 2009 KDIGO guidelines do not imply that the measurement of bone-derived turnover markers of collagen synthesis (such as P1CP) and breakdown (such as CTX, pyridinoline, or deoxypyridinoline) should be measured in patients with CKD 3-5D routinely (2C). Moreover, the 2016 draft clinical practice update of the KDIGO guideline does not introduce any changes to the bone turnover marker measurement [136].

The correction of biochemical abnormalities present in CKD patients accompanied by proper nutritional guidance and measures to prevent falls are of key importance to lower the risk of bone complications [7]. Studies concerning the impact of the consumption of particular food groups, macronutrients, and micronutrients on bone frailty, revealed that a protein intake of 1 g/kg in body weight per day should be fulfilled, except for patients with kidney disease, and vitamin deficiencies should be avoided in order to diminish the risk of frailty [137]. The occurrence of frailty has been linked to a higher risk of falls and fractures [138]. Therefore, it could be suggested that increased risk of fracture and frailty observed in CKD patients could be associated with inappropriate nutrition as well as the presence of bone mineral disorders. Proper management of CKD-MBD is targeted at the prevention of adverse consequences associated with secondary hyperparathyroidism. KDIGO guideline recommends treatment based on the serial measurement of surrogate markers of disordered mineral bone metabolism, including serum calcium, phosphate, intact parathyroid hormone, and 25-hydroxyvitamin D [139,140]. Hyperphosphatemia has also been demonstrated to be associated with adverse clinical outcomes in CKD patients, however, the normalization of phosphate levels with drugs does not seem to improve their outcomes. The administration of phosphate-lowering therapy in a clinical trial considerably reduced serum phosphate and insignificantly diminished serum level of FGF23, however, it aggravated coronary calcification scores in patients [141]. Therefore, KDIGO guidelines suggest that the prevention of hyperphosphatemia in patients with CKD stage G3a to G5D is much more essential than the normalization of phosphate levels or the lowering of its levels [140]. Daily phosphate intake should be limited to less than 800 mg through the control of the intake of high phosphate-containing products and carbonated beverages with phosphate additives [142]. Advanced stage CKD patients should be closely monitored to avoid malnutrition as most products rich in phosphate are also vital sources of protein. Phosphorus binders containing calcium (carbonate, acetate) can be prescribed for patients without hypercalcemia, but still, the calcium x phosphorus product and risk of ectopic calcification should be assessed. Protein restriction seems to be sufficient for keeping optimal phosphorus levels in predialysis patients, however, in those with hyperphosphatemia refractory to dietary measures the use of phosphorus binders is indicated [3,44]. In non-dialysis patients (GFR < 30 mL/min/1.73 m^2^), protein intake should be limited to 0.8 g/kg/day, with 50–60% of proteins of high biological value. Protein content in the diet cannot be too high as it could result in the accumulation of uremic toxins and hastening of renal damage but also it cannot be too low to avoid malnutrition, metabolic acidosis, loss of muscle mass, and bone fragility [3,7]. The intestinal absorptive capacity of various sources of phosphate should also be taken into consideration while making dietary recommendations, as the intestinal absorptive rate of inorganic phosphate contained in additives and beverages is 80%–100%, while in plant-based phosphate, it is 20 and 40% [143]. Moreover, in patients with CKD stages 3–4, it is recommended to maintain PTH levels below the upper normal limit of the norm [3] and serum calcium, phosphorus, and 25OHD concentrations within the normal range. Too high concentrations of PTH levels are associated with high turnover disease (osteitis fibrosa) resulting in an enhanced rate of bone resorption and fracture risk, while too low PTH level causes low turnover disease (adynamic bone disease) manifesting a greater risk of fractures due to low bone formation [3]. In CKD patients, adequate intake of phosphorus and protein, supplementation of vitamin D with pro-active (ergo or cholecalciferol) and/or active (calcitriol), as well as the use of phosphate binders, are frequently required [44]. Hypocalcemia in CKD patients can be alleviated with the use of calcium supplementation which also helps to diminish intestinal phosphorus absorption and to minimize the risk of hyperparathyroidism in this group [7,44]. However, in order to reduce the risk of extra-skeletal calcifications and to diminish the deleterious effect of calcium supplementation on the myocardium, especially in patients with diagnosed vascular calcification, the total amount of elemental calcium should not exceed 1.5 g per day, and it would be optimal if it was contained in the diet [44,144].

The supplementation of vitamin D (ergocalciferol or cholecalciferol) to correct hypovitaminosis D and abate hyperparathyroidism risk is recommended. However, in patients with severe renal impairment and reduced 1α-hydroxylase enzyme levels, the effectiveness of ergo or cholecalciferol replacement therapy is lower, and therefore the use of calcitriol may be sometimes required. Calcitriol enables the re-establishment of optimal calcium and phosphorus levels and decreases the risk of hyperparathyroidism, but at the same time it could increase the risk of hyperphosphatemia, hypercalcemia, vascular calcification, and adynamic bone disease thus it should be prescribed with caution [3,7,145].

## 5. Conclusions

Nutritional treatment of patients with advanced stages of CKD is aiming at prevention or correction of signs, symptoms of renal failure, avoidance of protein energy wasting (PEW), delaying or prevention of the occurrence of mineral/bone disturbances, and delaying the start of dialysis [146]. The results of studies suggest that progressive protein restriction is beneficial with the progression of renal insufficiency [147,148]; however, also other aspects of dietary management of CKD patients, including changes in sodium, phosphorus, and energy intake, as well as the source of protein and lipids (animal or plant origin) should be considered carefully. Energy intake must cover patients’ energy requirement (30–35 Kcal/Kg/day), in order to enable correct metabolic adaptation in the course of protein-restricted regimens and prevent negative nitrogen balance and protein-energy wasting [149]. Still, large clinical trials and population studies are required to confirm the impact of diets on CKD-MBD, frailty, and the risk of fractures observed in CKD patients. The analysis of molecular mechanisms of alterations induced by the use of an appropriate diet would also be of interest. However, the effects of nutritional interventions are difficult to assess due to the variability of dietary products.

Bone-targeted pharmacotherapy has been shown to bring hardly any effects in the field of fracture prevention. CKD patients differentially benefit from individualized classic therapy measures, such as the supplementation of vitamin D, phosphate level control with phosphate binders, the use of anti-resorptive agents, dialysis, and medical and surgical parathyroidectomy [64].

## Figures and Tables

**Table 1:** Study group.

Type of Study	Study Group	Most Important Results	Ref.
**Low Phosphate Diet**			
Randomized Controlled Trial	80 dialysis patients (experimental group: 41, control group: 39)	➢ Serum phosphorus decreased 1.67 mg/dL in the experimental group and 0.58 mg/dL in the control group (multivariate-adjusted difference 0.93 mg/dL; 95% CI 0.34–1.52; *p* = 0.003). Conclusions: Intensive dietary intervention targeted at phosphorus intake may be useful to decrease phosphorus retention and to ameliorate calcium-phosphorus metabolism in HD patients.	[74]
Review of 9 studies	634 participants	➢ Calcium-enriched bread increased serum calcium (MD −0.16 mmol/L, 95% CI −0.51 to −0.31), reduced serum phosphorus (53 participants: MD −0.41 mmol/L, 95% CI −0.51 to −0.31), and diminished the calcium × phosphate product (53 participants: MD −0.62 mmol^2^/L^2^, 95% CI −0.77 to −0.47); ➢ Low phosphorus intake reduced serum phosphorus (359 participants: MD −0.18 mmol/L, 95% CI −0.29 to −0.07; I(2) = 0%). Conclusions: limited low-quality evidence indicating that dietary interventions (calcium-enriched bread may positively influence CKD-MBD by increasing serum calcium, lowering serum phosphorus, the calcium × phosphate product, and FGF-23	[9]
2 × 2 factorial, single-blinded, placebo-controlled, 3-month study	39 patients with CKD stages 3 or 4 and normal serum phosphate levels randomly assigned to: (a) ad libitum diet + LC placebo (*n* = 10), (b) 900-mg phosphate diet + LC placebo (*n* = 10), (c) ad libitum diet + LC (*n* = 11), or (d) 900-mg phosphate diet + LC (*n* = 8)	➢ 900-mg phosphate diet compared with ad libitum diet—no significant reduction in FGF23 levels; ➢ LC alone compared with placebo—no significant reduction in FGF23 levels; ➢ Combined intervention significantly diminished FGF23 levels throughout the study period resulting in a 35% (95% Cl 8% to 62%) reduction by the end of study. Conclusions: Combination of LC plus counseling for a phosphate-restricted diet decreased FGF23 levels in patients with CKD stages 3–4 and normal serum phosphate levels.	[72]
Clinical trial. A quasi-experimental design.	63 dialysis (experimental group: 32, control group: 31). 20–30 min/month of additional diet education on monthly laboratory values and knowledge of dietary phosphorus management	➢ After 6 months, significantly higher gains in knowledge in the intervention group, and considerably lower serum phosphorus and calcium/phosphorus product levels (*p* < 0.01) than in the control group. Conclusion: Patients who received extra education monthly showed positive changes, which may be beneficial in reducing hyperphosphatemia.	[75]
Clinical trial. Educational intervention and one-to-one teaching session with renal dietitian	56 stable adult hemodialysis patients with hyperphosphatemia	➢ Significantly decreased serum phosphate after the education session in the intervention group compared to patients’ previous results; ➢ Improved results were sustained over a period of 3 months. Conclusion: Dietetic educational intervention can favorably alter patients’ serum phosphate levels, with potential impact on morbidity and mortality.	[76]
**Low-Protein Diets and Supplemented Very-Low-Protein Diets**			
Review of 9 studies	634 participants	➢ Very low protein intake was not superior to conventional low protein intake in terms of effect on serum phosphorus (41 participants: MD −0.12 mmol/L, 95% CI −0.50 to 0.25), serum calcium (MD 0.00 mmol/L, 95% CI −0.17 to 0.17), or alkaline phosphatase (MD −22.00 U/L, 95% CI −78.25 to 34.25); ➢ PTH was significantly lower in the very low protein intake group (41 participants: MD −69.64 pmol/L, 95% CI −139.83 to 0.54). Conclusions: limited low-quality evidence indicating that dietary interventions (low phosphorus/protein intake) may positively affect CKD-MBD by increasing serum calcium, decreasing serum phosphorus, the calcium × phosphate product and FGF-23.	[9]
Clinical trial	22 with CRF randomly assigned to a conventional low-protein diet (0.6 g protein/kg/day) or a very-low-protein diet (0.4 g protein/kg/day) supplemented with essential AA	➢ No significant changes in body mass index, arm muscle area, percentage body fat, serum albumin, and transferrin levels in any of the groups; ➢ Renal function (measured on the basis of serum creatinine) stabilized in both groups during intervention, with no significant difference between the groups; ➢ No significant changes and no significant differences between the groups in serum levels of parathyroid hormone and alkaline phosphatase, urine cyclic adenosine monophosphate, tubular reabsorption of phosphate, and the theoretical renal threshold for phosphate. Conclusions: Supplemented very-low-protein diet was not superior to the conventional low-protein diet in terms of its effect on protein-energy status, renal function, and biochemical parameters of renal osteodystrophy.	[93]
Comparative study	12,812 subjects assigned to (a) < 0.8 g/kg/day, (b) 0.8–1.0 g/kg/day, (c) 1.0–1.2 g/kg/day, and (d) ≥ 1.2 g/kg/day).	➢ Statistically significant differences in bone mineral density (BMD) or T scores (*p* < 0.0001)—the higher the protein intake, and the higher the bone BMD and T scores (*p* < 0.0001); ➢ Higher protein diets were associated with higher BMD in the femoral neck, trochanter, intertrochanteric, and total femoral areas (*p* = 0.032, 0.0036, 0.008, and 0.0039, respectively); ➢ Such increased BMD benefits of a higher protein diet were not observed in CKD patients. Conclusions: Higher protein diets resulted in higher femoral BMD only in subjects without CKD. CKD was found to be a risk factor for low BMD in the intertrochanteric bone region.	[94]
Meta-analysis of 17 RCTs	1459 participants	➢ Restricted protein diet with KAs significantly preserved eGFR and decreased proteinuria, serum phosphate, parathyroid hormone (PTH) level, systolic BP, diastolic BP, and serum cholesterol; ➢ VLPD with KAs could be superior to LPD with KAs in slowing the decline in eGFR; ➢ Only VLPD with KAs considerably improved serum PTH, systolic and diastolic BP; ➢ Only LPD with KAs considerably increased serum albumin and serum calcium. Conclusion: Restricted protein diet with KAs could effectively improve kidney endpoints including preserving eGFR and diminishing proteinuria, BP levels, and CKD-mineral bone disorder parameters without causing malnutrition. VLPD with KAs seems to be more effective in reducing the rate of eGFR worsening, decreasing BP, diminishing serum PTH, and less in raising serum calcium levels.	[100]
A randomized, controlled pilot study	79 non-diabetic CKD 3b-4 stage patients79 non-diabetic CKD 3b-4 stage patients	➢ LPD group displayed body mass indices’ decrease (*p* = 0.046) (muscle body mass loss in men (*p* = 0.027) and woman (*p* = 0.044)) after 14 months; ➢ In LPD + KA group FGF-23 was lower (*p* = 0.029), and Klotho was higher (*p* = 0.037) compared to LPD one; ➢ The increase in AI (*p* = 0.034), VCS (*p* = 0.048), and LVMMI (*p* = 0.023) was reported more often in the LPD group at the end of study; Conclusion: LPD + KA is associated with nutritional and more efficient correction of FGF-23 and Klotho abnormalities leading to reduced cardiovascular calcification and cardiac remodeling in CKD. Prolonged application of LPD alone may lead to malnutrition.	[97]
A systematic review and meta-analysis	End-stage renal disease patients	➢ Low-protein diet (LPD) supplemented with KA, compared with normal protein diet, could improve serum albumin (*p* < 0.00001), hyperparathyroidism (*p* < 0.00001) and hyperphosphatemia (*p* = 0.008). ➢ No differences in triglyceride, cholesterol, hemoglobin, Kt/v, and CRP were observed between different protein intake groups. Conclusion: Restricted protein diet supplemented with KA may improve nutritional status and prevent hyperparathyroidism in ESRD patients.	[101]
Prospective, randomized, crossover controlled trial	60 patients with CKD grades 3B-4 assigned to: (a) 3 months of free diet (FD), 6 months of VLPD, 3 months of FD and 6 months of MD; and (b) 3 months of FD, 6 months of MD, 3 months of FD and 6 months of VLPD	➢ MD and VLPD was associated with lower diastolic BP pressure and reduced serum levels of urea, sodium, phosphorus, and parathyroid hormone, as well as higher serum levels of bicarbonate and hemoglobin; ➢ Both MD and VLPD, when compared with FD, were related to reduction in serum Hcit levels and Hcit/Lys ratios (*p* < 0.001). Conclusion: Nutritional treatments which significantly diminished serum levels of urea are associated with reduced protein carbamylation.	[102]
A prospective, randomized, controlled crossover study	32 patients randomized into: (a) very-low-protein diet (0.3 g/kg body wt per day) supplemented with KA (1st week) and a low-protein diet (2nd week), or (b) a low-protein diet (1st week) and a very-low-protein diet (2nd week)	➢ One week of the very-low-protein diet was associated with a decrease in FGF23 levels (33.5%), serum phosphate (12%), and urinary phosphate (34%) compared with the low-protein diet; ➢ Serum and urinary phosphate levels and protein intake were significant determinants of FGF23 (95% Cl 1.04 to 1.19, 1.12 to 1.37, and 1.51 to 2.23, respectively). Conclusions: A very-low-protein diet supplemented with ketoanalogs reduced fibroblast growth factor 23 levels in CKD patients not yet on dialysis.	[91]
A post hoc analysis of the MDRD Study	CKD patients receiving a usual-protein (UP) or low-protein (LP) diet in study A or an LP or very LP (VLP) with ketoacids diet in study B	➢ Dietary protein restriction effectively reduced urinary phosphate levels and was associated with a very modest but sustained decrease in serum phosphate levels; ➢ Patients with mild CKD achieved stable serum phosphate levels over 3 years.	[92]
Clinical trial	Patients with early (*n* = 15) and advanced (*n* = 20) CKD	➢ Decreased serum FGF23 levels in both groups; ➢ Changes in FGF23 levels correlated with changes in 24 h urinary phosphorus excretion in the advanced CKD group; ➢ Decreased serum intact parathyroid hormone levels only in the advanced CKD group; ➢ Increased serum 1,25-dihydroxyvitamin D levels only in the early CKD group. Conclusions: Consuming a standard low-protein diet decreased serum FGF23 levels in patients with CKD. Serum FGF23 levels may therefore be a useful marker to monitor the effects of a low-protein diet in early and advanced stage CKD.	[99]
Clinical trial	40 MHD patients with uncontrolled hyperphosphatemia randomized into either low sLP or NP group for 8 weeks. After 8 weeks, the sLP group was shifted to NP for another 8 weeks.	➢ Significantly decreased serum phosphorus level and calcium-phosphate product at the end of the first 8 weeks in the sLP group vs. the basal value and the NP group (*p* < 0.001); ➢ No difference in C-reactive protein, Kt/V, and nutritional index. Conclusion: Short-term restriction of DPI supplemented with keto acids could decrease hyperphosphatemia and calcium-phosphate product, while keeping stable nutritional status among MHD patients.	[104]
	13 stable patients with GFR 15 ± 5 mL/min receiving a VLPD (0.3 g/kg/day protein) supplemented with AA and KAs	➢ GFR, albumin, and pre-albumin levels remained stable; ➢ Decreased urea urinary excretion at 3 months and then slightly increased at 2 years; ➢ No significant change in total fat mass or percent fat mass; ➢ Total bone mass, lumbar or hip site bone mass, and Z-score significantly decreased from T0 to 1 and 2 years (*p* <0.05). Conclusion: A supplemented VLPD is nutritionally safe for a long period, but attention must be paid to bone mass.	[106]
**High-Protein Diets in CKD and ESKD**			
Observational study	Women (*n* = 39,066) and men (*n* = 31,149) from UK Biobank (participants aged 40–69 years)	➢ Positive and linear association between protein intake and BMD in women (β-coefficient 0.010 [95% CI 0.005; 0.015, *p* < 0.0001]) and men (β-coefficient 0.008 [95% CI 0.000; 0.015, *p* = 0.044]). Conclusions: Higher protein intakes are positively associated with BMD in both men and women. This indicates that higher protein intakes may be beneficial for both men and women.	[111]
A post-hoc analysis of the IHOPE trial	HD patients (138) randomized for 12 months to: placebo (CON), protein supplementation (PRO), or protein + exercise training (PRO + EX).	➢ Patients ≥ 60 y on protein supplementation maintained hip bone mineral density (h-BMD); ➢ Decrease in h-BMD in placebo group (placebo 0.87 ± 0.13 and 0.84 ± 0.13 vs. supplementation 0.87 ± 0.16 and 0.86 ± 0.16 g/cm^2^ at 0 and 12 months, respectively; *p* = 0.02); ➢ Similar trend observed for the femoral neck BMD (*p* = 0.07); ➢ Lac of such effect in patients < 60 y (*p* = 0.826); ➢ No effect of protein supplementation on body composition or blood markers of bone metabolism (calcium, phosphorus, and parathyroid hormone) in either age group (*p* > 0.05). Conclusions: Intradialytic protein supplementation attenuated the decrease in h-BMD, a predictor of fractures, in older HD patients.	[112]
**Plant-Based Diets**			
A crossover randomized trial	9 patients (mean eGFR 32 mL/min); vegetarian vs. meat diets	➢ 1 week of a vegetarian diet lowered serum phosphorus levels and decreased FGF23 levels; ➢ Significant differences in diurnal variation for blood phosphorus, calcium, PTH, and urine fractional excretion of phosphorus between the vegetarian and meat diets; ➢ Strong correlation between 24-h fractional excretion of phosphorus and a 2-h fasting urine collection in those on vegetarian diet only. Conclusions: Source of protein significantly affects phosphorus homeostasis in patients with CKD.	[119]
An observational study	13 subjects with CKD 3–4; omnivorous diet containing 70% protein from plants for 4 weeks	➢ Significantly decreased by 215 ± 232 mg/day (*p* < 0.001) urine phosphorus (after 4 weeks); ➢ No significant changes in serum FGF23, phosphorus, or PTH; ➢ Markedly reduced urine sodium and titratable acid; Conclusions: A 70% plant protein diet is safe, tolerated, and efficacious in lowering urine phosphorus excretion and may be an alternative to phosphate binders.	[120]
A randomized crossover trial	15 patients with CRF; soya-based vegetarian low-protein diet (VPD) and an animal-based low-protein diet (APD) for 6 months.	➢ Unchanged mean GFR filtration after 6 months on each diet; ➢ Slower rate of fall of glomerular filtration; ➢ Similar nutritional status (body mass index, midarm circumference, and lean body mass and percent body fat), serum transferrin, cholesterol and albumin, and total lymphocyte count on the two diets; ➢ Lower blood urea nitrogen, urine urea nitrogen, protein catabolic rate, and 24-h urine creatinine and phosphate on the VPD compared to APD; ➢ Both diets slowed down the progression of CRF. Conclusions: VPD is well tolerated in CRF and is associated with lower protein and phosphate intakes and a higher caloric intake than an APD and may, therefore, be used as a safe alternative or partial substitute for the usual APD in CRF.	[121]

## Data Availability

Not applicable.
